# Innovation and optimization in autoimmune encephalitis trials: the design and rationale for the Phase 3, randomized study of satralizumab in patients with NMDAR-IgG-antibody-positive or LGI1-IgG-antibody-positive autoimmune encephalitis (CIELO)

**DOI:** 10.3389/fneur.2024.1437913

**Published:** 2024-08-13

**Authors:** Soon-Tae Lee, Hesham Abboud, Sarosh R. Irani, Hideto Nakajima, Amanda L. Piquet, Sean J. Pittock, E. Ann Yeh, Jiawei Wang, Sharmila Rajan, James Overell, Jillian Smith, Jane St Lambert, Muna El-Khairi, Marina Gafarova, Jeffrey M. Gelfand

**Affiliations:** ^1^Department of Neurology, Seoul National University Hospital, Seoul National University College of Medicine, Seoul, Republic of Korea; ^2^Department of Neurology, University Hospitals Cleveland Medical Center, Cleveland, OH, United States; ^3^Oxford Autoimmune Neurology Group, Nuffield Department of Clinical Neurosciences, University of Oxford, Oxford, United Kingdom; ^4^Departments of Neurology and Neurosciences, Mayo Clinic, Jacksonville, FL, United States; ^5^Division of Neurology, Department of Medicine, Nihon University School of Medicine, Tokyo, Japan; ^6^Department of Neurology, University of Colorado, Aurora, CO, United States; ^7^Department of Neurology, Mayo Clinic College of Medicine, Rochester, MN, United States; ^8^Division of Neurology, Department of Pediatrics, The Hospital for Sick Children, University of Toronto, Toronto, ON, Canada; ^9^Department of Neurology, Beijing Tongren Hospital, Capital Medical University, Beijing, China; ^10^Product Development Neuroscience, Genentech, Inc., South San Francisco, CA, United States; ^11^Product Development Neuroscience, F. Hoffmann-La Roche Ltd., Basel, Switzerland; ^12^Roche Products Ltd., Welwyn Garden City, United Kingdom; ^13^Department of Neurology, UCSF Weill Institute for Neurosciences, San Francisco, CA, United States

**Keywords:** autoimmune encephalitis (AIE), clinical trial, leucine-rich glioma-inactivated 1 (LGI1), *N*-methyl-d-aspartic acid receptor (NMDAR), satralizumab

## Abstract

**Background:**

Autoimmune encephalitis (AIE) encompasses a spectrum of rare autoimmune-mediated neurological disorders, which are characterized by brain inflammation and dysfunction. Autoantibodies targeting the *N*-methyl-d-aspartic acid receptor (NMDAR) and leucine-rich glioma-inactivated 1 (LGI1) are the most common subtypes of antibody-positive AIE. Currently, there are no approved therapies for AIE. Interleukin-6 (IL-6) signaling plays a role in the pathophysiology of AIE. Satralizumab, a humanized, monoclonal recycling antibody that specifically targets the IL-6 receptor and inhibits IL-6 signaling, has demonstrated efficacy and safety in another autoantibody-mediated neuroinflammatory disease, aquaporin-4 immunoglobulin G antibody-positive neuromyelitis optica spectrum disorder, and has the potential to be an evidence-based disease modifying treatment in AIE.

**Objectives:**

CIELO will evaluate the efficacy, safety, pharmacodynamics, and pharmacokinetics of satralizumab compared with placebo in patients with NMDAR-immunoglobulin G antibody-positive (IgG+) or LGI1-IgG+ AIE.

**Study design:**

CIELO (NCT05503264) is a prospective, Phase 3, randomized, double-blind, multicenter, basket study that will enroll approximately 152 participants with NMDAR-IgG+ or LGI1-IgG+ AIE. Prior to enrollment, participants will have received acute first-line therapy. Part 1 of the study will consist of a 52-week primary treatment period, where participants will receive subcutaneous placebo or satralizumab at Weeks 0, 2, 4, and every 4 weeks thereafter. Participants may continue to receive background immunosuppressive therapy, symptomatic treatment, and rescue therapy throughout the study. Following Part 1, participants can enter an optional extension period (Part 2) to continue the randomized, double-blind study drug, start open-label satralizumab, or stop study treatment and continue with follow-up assessments.

**Endpoints:**

The primary efficacy endpoint is the proportion of participants with a ≥1-point improvement in the modified Rankin Scale (mRS) score from study baseline and no use of rescue therapy at Week 24. Secondary efficacy assessments include mRS, Clinical Assessment Scale of Autoimmune Encephalitis (CASE), time to rescue therapy, sustained seizure cessation and no rescue therapy, Montreal Cognitive Assessment, and Rey Auditory Verbal Learning Test (RAVLT) measures. Safety, pharmacokinetics, pharmacodynamics, exploratory efficacy, and biomarker endpoints will be captured.

**Conclusion:**

The innovative basket study design of CIELO offers the opportunity to yield prospective, robust evidence, which may contribute to the development of evidence-based treatment recommendations for satralizumab in AIE.

## Introduction

Autoimmune encephalitis (AIE) encompasses a spectrum of rare, autoimmune-mediated neurological disorders and is characterized by brain inflammation and dysfunction (1–4). People with AIE may experience a wide range of debilitating, and potentially life-threatening symptoms, including cognitive impairment, seizures, memory deficits, psychiatric symptoms, movement disorders, and altered mental status, which may only improve partially with standard immunotherapy and may lead to long-term neurological disability (1, 5–14). Although the closely related group of disorders that encompass AIE have shared overlapping clinical features and neuroimaging findings, they are differentiated by distinct pathophysiological mechanisms, including the presence or absence of neuronal autoantibodies that drive inflammation and synaptic dysfunction in the central nervous system (CNS), as well as characteristic clinical syndromes and epidemiologic characteristics (1–3, 15, 16).

Autoantibodies targeting the *N*-methyl-d-aspartic acid receptor (NMDAR) and leucine-rich glioma-inactivated 1 (LGI1) comprise the most common subtypes of antibody-positive AIE (2, 17). NMDAR immunoglobulin G antibody-positive (NMDAR-IgG+) AIE commonly affects children and younger adults (2). The primary clinical presentations include psychiatric symptoms, memory loss, seizures, speech disturbance, dyskinesia (typically, orofacial and limb dyskinesia), decreased consciousness, and autonomic dysfunction (2, 11, 18, 19). Up to 40% of patients with NMDAR-IgG+ AIE have an ovarian teratoma and, in these cases, it is thought that germinal centers within the tumor and cervical lymph nodes generate autoantibodies that are misdirected against host antigens in the CNS (20–22). LGI1-IgG+ AIE generally affects middle-aged to older patients, and is associated with frequent faciobrachial dystonic seizures that are characterized by stereotyped dystonic jerks in the face, arm or leg; localization-related seizures; rapidly progressive cognitive impairment with amnesia; and hyponatremia (12, 23–25). AIE may be autoantibody seronegative (26), or may be linked to various other antibodies targeting proteins, such as contactin-associated protein-like 2, γ-aminobutyric acid receptors type A or B, dipeptidyl-peptidase-like protein 6, and glutamic acid decarboxylase 65, each of which is associated with specific phenotypes and different levels of systemic cancer risk (27).

There are currently no approved disease-modifying therapies specifically for AIE, and treatment relies on the off-label use of existing therapies (28–30). Acute immunotherapy regimens commonly include glucocorticoids, intravenous immunoglobulin, plasma exchange therapy, or a combination of these (11, 14, 15, 28–31). In patients with refractory AIE, additional immunosuppressive therapy may involve rituximab, cyclophosphamide, tocilizumab, mycophenolate mofetil, azathioprine, bortezomib, daratumumab, tofacitinib, or low-dose IL-2 (14, 15, 28, 30–43). The current treatment approach for AIE has numerous limitations that highlight the need for higher quality, lower risk of bias evidence, particularly that generated by prospective, multicenter, randomized controlled trials, to guide treatment choices in AIE to address acute and long-term effects (2, 15, 28, 30).

Approximately 45% of patients with NMDAR-IgG+ AIE have moderate functional neurological impairments (modified Rankin scale [mRS] score 2–3) >2 years after disease onset (after the acute disease stage) (44). After almost 5 years following disease onset, 65% of patients with NMDAR-IgG+ AIE have moderate-to-severe cognitive deficits (composite score 2–4 [composite score of cognitive impairment was defined as the number of affected domains on tests of working memory, verbal and visual episodic memory, executive function, and attention]) despite improvements in functional neurological outcomes (44). In LGI1-IgG+ AIE, approximately 12–40% of patients experience relapses even after achieving remission with immunotherapy (45–47). Many patients with NMDAR-IgG+ and LGI1-IgG+ AIE continue to experience long-term impairments, failing to regain their cognitive and functional status (14, 21, 46, 48–54). This is often attributed to delayed treatment and the relatively slow or incomplete response to currently available medications, leading to ineffective control of CNS inflammation (25, 54–56). Consequently, there is an urgent need for prospectively generated high-quality evidence in AIE to guide treatment choices and to inform treatment paradigms to address both the acute and long-term effects of this rare neurological disorder.

Innovations in clinical trial designs have resulted in the development of master protocols to address unmet needs and to answer multiple clinical questions more efficiently (57). One example is the basket study design (57). The basket study design aims to evaluate a single intervention in multiple diseases or disease subtypes (57) and has been used to improve trial efficiency in both oncology (58, 59) and non-malignant conditions (60, 61). Within a basket study, each disease-specific arm (in this case, NMDAR-IgG+ and LGI1-IgG+ AIE) is analyzed independently. Each arm has outcome measures tailored to its specific disease subtype. This results in improved interpretability and reliability of study data, while leveraging the shared infrastructure and efficiencies of the master basket study protocol. AIE is particularly suited to a basket study design, given that the different autoantibody subtypes have distinct epidemiologic characteristics and pathologies while sharing other similarities.

CIELO (NCT05503264) is a Phase 3, randomized, placebo-controlled, basket study of satralizumab, an interleukin-6 (IL-6) receptor (IL-6R) inhibitor, in patients with NMDAR-IgG+ or LGI1-IgG+ AIE. IL-6 is a pro-inflammatory cytokine with pleiotropic functions, such as T-cell polarization toward an inflammatory T-helper 17 phenotype, promotion of survival and functioning of autoantibody-producing plasma cells, and blood–brain barrier disruption (62, 63). In NMDAR-IgG+ and LGI1-IgG+ AIE, processes regulated by IL-6 signaling (e.g., B- and T-cell differentiation, B-cell proliferation, and regulation of the blood–brain barrier) have been suggested to have a pathogenic role (11, 62, 64–67), and IL-6 levels are elevated in the cerebrospinal fluid (CSF) of patients with NMDAR-IgG+ AIE (68–71) and in the serum of patients with LGI1-IgG+ AIE (72). Through IL-6R inhibition, satralizumab has the potential to modulate key upstream immunopathogenic mechanisms in AIE (62, 63, 73). Retrospective studies and a case report of IL-6R inhibition have been described in patients with NMDAR-IgG+ and LGI1-IgG+ AIE, with observed clinical improvements (40, 41, 74).

CIELO is the first study of satralizumab, a humanized, immunoglobulin G2 (IgG2), monoclonal recycling antibody against the IL-6R (75), in AIE. Satralizumab provides durable inhibition of IL-6 signaling (76) and is indicated for another autoantibody-mediated neuroimmunological disease, aquaporin-4 immunoglobulin G antibody-positive neuromyelitis optica spectrum disorder, as monotherapy or as an add-on to immunosuppressive therapy (IST) (75, 77–80). As such, satralizumab has the potential to become the first evidence-based IL-6 signaling inhibition treatment in AIE.

Design and execution of studies in AIE and other rare diseases present inherent challenges. These include difficulties related to the heterogeneity of the patient population and symptoms, identification of eligible participants, appropriately powered sample size, use of placebo, availability of rescue therapy, disease outcome definitions, trial duration, and selection of clinically meaningful endpoints (81, 82). Anticipating these challenges ahead of study design can increase the likelihood of the successful generation of robust data in the field. In the last decade, nine randomized clinical trials in AIE have been initiated; six are ongoing and three have been terminated (Table 1) (29, 82–90).

**Table 1:** Randomized clinical trials in AIE.

RCT	Intervention(s)	Status	Design	Population	Primary treatment period	Primary endpoint(s)	Extension period
**Basket study designs in NMDAR-IgG+ and LGI1-IgG+ AIE**							
NCT05503264 (CIELO) (83)	**Drug: Satralizumab** Other: Placebo	Recruiting; North America, South America, Europe, and Asia	Phase 3, randomized, double-blind, placebo-controlled, multicenter, basket study	**Adolescent and adult population (≥12 years old)** NMDAR-IgG+ and LGI1-IgG+ AIE (adults only) New onset^a^ or an incomplete responder^b^	52 weeks	mRS score improvement ≥1 from baseline and no use of rescue therapy at Week 24	≥2 years. Participants can choose from one of three options: (1) continue on the randomized, double-blind study drug, (2) start open-label satralizumab, or (3) stop study treatment and continue with follow-up assessments
**Studies including participants with NMDAR-IgG+ AIE only**							
NCT04372615 (ExTINGUISH) (84)	**Drug: Inebilizumab** Other: Placebo	Recruiting; United States, The Netherlands, and Spain	Phase 2b, randomized, double-blind, placebo-controlled study	**Adolescent and adult population (≥12 years old)** NMDAR-IgG+ AIE Prior treatment with methylprednisolone, PLEX, and/or IVIg	96 weeks	Change in mRS score from baseline to Week 16 and number of AEs and SAEs at Week 96	N/A
**Studies including participants with LGI1-IgG+ AIE only**							
NCT04875975 (85)	**Drug: Rozanolixizumab** Other: Placebo	Terminated due to enrollment challenges	Phase 2, randomized, double-blind, placebo-controlled, multicenter study	LGI1-IgG+ AIE (18–89 years old) Onset of disease symptoms ≤12 months prior to screening	25 weeks	Seizure freedom at Week 25	N/A
**Studies including participants with a range of AIE subtypes**							
NCT03835728 (82)	**Drug: Ocrelizumab** Other: Placebo	Terminated due to failure to reach target enrollment	Phase 2, randomized, double-blind, placebo-controlled study	NMDAR-IgG+, LGI1-IgG+, CASPR2-IgG+ or DPPX-IgG+ AIE (>18 years old) New diagnosis of AIE	12 months	Confirmed clinical worsening^c^ within 12 months. Data published: Two patients were randomly assigned to treatment (one patient [with NMDAR-IgG+ AIE] had clinical improvement and one patient [with LGI1-IgG+ AIE] remained clinically stable). One patient (with NMDAR-IgG+ AIE) was randomly assigned to placebo (met the primary endpoint at 12 weeks and received open-label ocrelizumab)	N/A
NCT03194815 (SINAPPS2) (86)	**Drug: IVIg and rituximab** Other: Placebo	Recruiting; United Kingdom	Phase 2a, randomized, double-blind, placebo-controlled, multicenter study	AIE (18–70 years old)	12 months (up to 18 months if required)	Time to symptomatic recovery^d^ of psychosis up to 18 months	N/A
NCT03993262 (Generate-Boost) (87)	**Drug: Bortezomib** Other: Placebo	Recruiting; Germany	Phase 2, randomized, double-blind, investigator-initiated, placebo-controlled, multicenter study	Severe^e^ AIE (>18 years old) Prior treatment with rituximab	17 weeks	mRS score at Week 17	N/A
NCT05177939 (88)	**Drug: IVIg** Other: Methyl-prednisolone (active comparator)	Recruiting; Japan	Phase 3, randomized, active-controlled study	**Adult and pediatric population (≥15 years old)** AIE refractory to pulse therapy Use of IVIg therapy and steroid pulse therapy	12 weeks	CASE responder^f^ at Week 4	N/A
NCT02697292 (29, 89)	**Drug: IVIg** Other: Placebo	Terminated due to failure to reach target enrollment	Phase 3, randomized, double-blind, placebo-controlled study	LGI1-IgG+, CASPR2-IgG+ or VGKC-IgG+ AIE (18–80 years old) Duration of epilepsy <3 years	5 weeks	50% reduction in seizure frequency from baseline to Week 5 Data published: 6 of 8 patients in the IVIg group and 2 of 9 patients in the placebo group reached the primary clinical endpoint, which was statistically significant (29)	N/A
NCT03542279 (90)	**Drug: IVIg and high-dose glucocorticoid (early PLEX)** Other: IVIg and high-dose glucocorticoid (active comparator; non-early PLEX)	Recruiting; China	Prospective, randomized, controlled trial	**Adult and pediatric AIE population (14–65 years old)**	24 months	mRS score at Month 3	N/A

^a^Participants were classified as new onset if they started their first acute first-line therapy ≤ 8 weeks prior to randomization and received no prior immunotherapy additional to acute first-line therapy. ^b^Participants were classified as incomplete responders if they initiated their first acute first-line therapy > 8 weeks prior to randomization and received immunotherapy beyond first acute first-line therapy. ^c^Clinical worsening was defined as a perception of decline reported by the patient, caregiver or clinician or a decrease in the Lawton and Brody IADL by ≥ 1 points. Additionally, one of the following criteria was required to confirm worsening: (1) a documented worsening of the TFLS by 0.5 standard deviations or greater or (2) clinical symptoms consistent with encephalitis requiring hospitalization. ^d^Symptomatic recovery is defined as a score of ≤ 3 on each of the following PANSS items: Positive Scale items (P1, delusions; P2, conceptual disorganization; P3, hallucinatory behavior), negative Scale items (N1, blunted affect; N4, passive/apathetic social withdrawal; N6, lack of spontaneity) and items from the General Pathology Scale (G5, mannerisms/posturing; G9, unusual thought content) sustained for ≥ 6 months. ^e^Severe AIE is defined as mRS ≥ 3. ^f^CASE responder is defined as a patient whose CASE score at Week 4 of the post-treatment follow-up period after treatment with investigational product improved by ≥ 40% compared to the pretreatment period.

AE, adverse event; AIE, autoimmune encephalitis; CASE, Clinical Assessment Scale in Autoimmune Encephalitis; CASPR2-IgG+, contactin-associated protein-like 2 immunoglobulin G antibody-positive; DPPX-IgG+, dipeptidyl-peptidase-like protein-6 immunoglobulin G antibody-positive; IADL, Instrumental Activities of Daily Living Scale; IgG, immunoglobulin G; IVIg; intravenous immunoglobulin; LGI1-IgG+, leucine-rich glioma-inactivated 1 immunoglobulin G antibody-positive; mRS, modified Rankin Scale; N/A, not applicable; NMDAR-IgG+, *N*-methyl-D-aspartic acid receptor immunoglobulin G antibody-positive; PANSS, Positive and Negative Syndrome Scale; PLEX, plasma exchange; RCT, randomized controlled trial; SAE, serious adverse event; TFLS, Texas Functional Living Scale; VGKC-IgG+, voltage-gated potassium channel immunoglobulin G antibody-positive.Bold text was used to differentiate some of the text.

CIELO is a uniquely designed basket study that aims to address the common study design challenges faced in rare disease trials by aiming to balance the interests of patients with the urgent need for scientific advancement in this field. Here, we discuss the crucial trial design and execution decisions, including the learnings from previous and ongoing AIE studies, which may significantly differentiate CIELO from other trials in AIE.

## Methods and analysis

### Objectives

The primary objective of this study is to evaluate the efficacy, safety, pharmacodynamics (PD), and pharmacokinetics (PK) of satralizumab compared with placebo in patients with NMDAR-IgG+ or LGI1-IgG+ AIE in a randomized, double-blind period. The long-term safety and efficacy of satralizumab will be assessed over a treatment exposure period of ≥3 years, during the double-blind and then optional extension period.

### Study design

**Figure 1:**
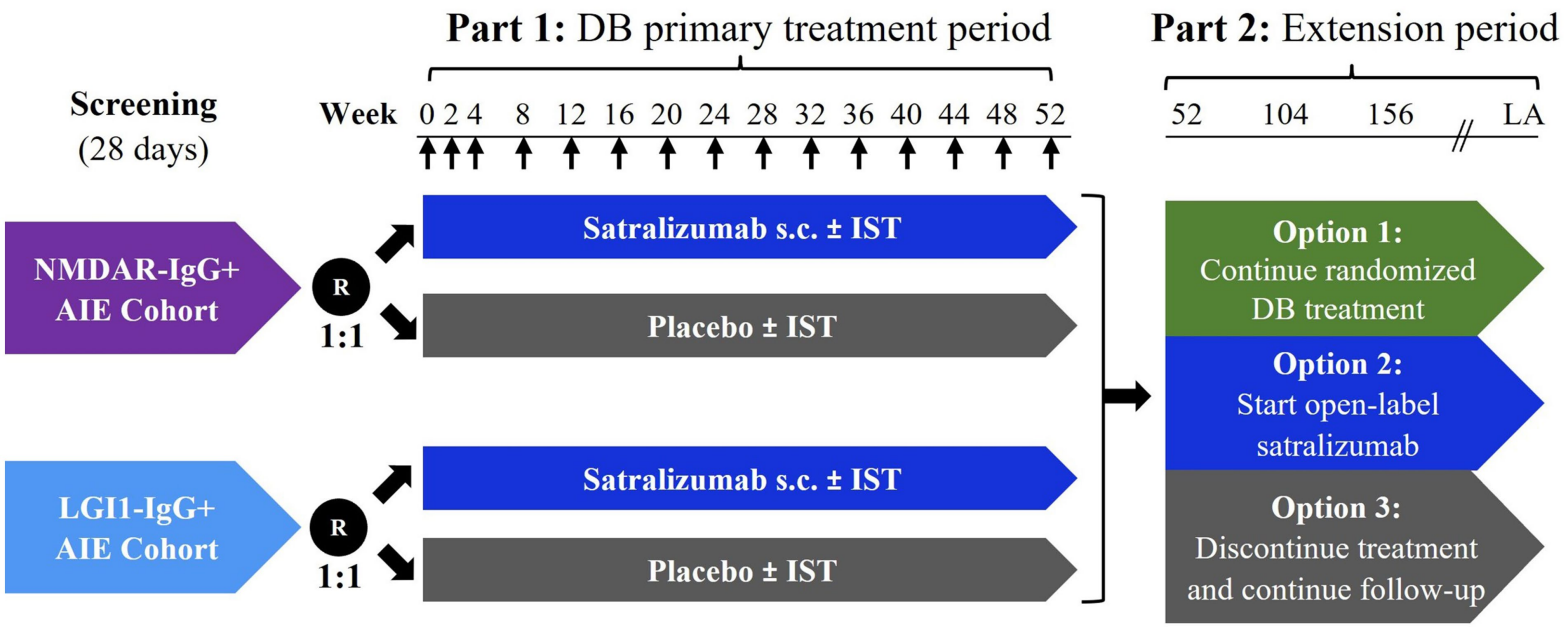
Study schema. ↑ Administration of subcutaneous satralizumab or placebo. Week 0 of Part 2 (extension period) coincides with Week 52 of Part 1 (primary treatment period). Participants who choose to start open-label satralizumab treatment and were treated with active drug in Part 1 will be administered a placebo dose at extension Week 2 to maintain blinding of treatment assignment in Part 1. The duration of Part 2 will be approximately 2 years after the last participant begins Part 2. AIE, autoimmune encephalitis; DB, double-blind; IST, immunosuppressive therapy; LA, last administration; LGI1-IgG+, leucine-rich glioma-inactivated 1 immunoglobulin G antibody-positive; NMDAR-IgG+, *N*-methyl-d-aspartic acid receptor immunoglobulin G antibody-positive; R, randomization; s.c., subcutaneous.

CIELO is a Phase 3, randomized, double-blind, placebo-controlled, multicenter, basket study of satralizumab in patients with NMDAR-IgG+ and LGI1-IgG+ AIE (Figure 1). The study consists of two parts. In Part 1, participants will receive placebo or satralizumab over a period of 52 weeks. In Part 2, participants have the option to continue for an extension period, lasting approximately 2 years after the last patient begins Part 2.

To avoid recruitment challenges reported by single-center trials in AIE (29, 82), CIELO will be conducted at approximately 85 sites in 15 countries, including countries in Asia, Europe, North America, and South America, maximizing the likelihood of identifying eligible participants and improving potential global generalizability. The study will enroll approximately 152 participants with NMDAR-IgG+ or LGI1-IgG+ AIE.

The basket design offers a unique opportunity to assess whether satralizumab is efficacious in these distinct patient subtypes and to what extent; this will be particularly valuable in AIE, which currently does not have subtype-specific treatment guidelines despite its heterogeneity.

### Study population and analysis groups

The heterogeneity of AIE is challenging for trial design because it may affect the interpretability and reliability of study data. Regulators have provided guidance about being as specific as possible with disease subtypes and the use of separate studies or arms for different subtypes of AIE. The basket design allows each cohort to have its own placebo arm and be analyzed separately with independent Type I error control at a two-sided 5% significance level.

Given the clear unmet need in both newly diagnosed AIE patients and those with incomplete response to prior immunosuppressive medications, as well as evidence suggesting the potential benefit of IL-6R blockade in both populations (40, 41, 91), participants will be subclassified as “new onset” or “incomplete responders” (Table 2). Although differential efficacy is not expected, this will facilitate the potential identification of any additional findings that may advance the understanding of this complex disease and increase the eligibility and recruitment to provide a balanced representation of the real-world AIE population.

**Table 2:** Definitions of new onset and incomplete responder for inclusion criteria.

New onset	Incomplete responder
**Stable (for at least 24 h) mRS score ≥ 2, measured at study baseline**	
Started first acute first-line therapy ≤8 weeks prior to randomization *and* received no prior immunotherapy additional to acute first-line therapy	Started first acute first-line therapy >8 weeks prior to randomization *and* received immunotherapy beyond first acute first-line therapy^a^

^a^Rituximab, ISTs, oral glucocorticoids, or repeated pulse therapy.

IST, immunosuppressive therapy; mRS, modified Rankin Scale.

### Eligibility criteria and recruitment

#### Cohort 1: Definite and probable NMDAR-IgG+ AIE

Eligible participants for the NMDAR-IgG+ AIE cohort will be ≥12 years old with a diagnosis of NMDAR-IgG+ AIE according to clinical criteria adapted from Graus et al. (1). In some clinical practice settings, access to NMDAR-IgG assays, and specifically CSF sampling for NMDAR-IgG, can be inconsistent, delayed, or difficult to achieve (9, 92). Hence, this study will include both definite NMDAR-IgG+ AIE (NMDAR-IgG-seropositivity confirmed via a cell-based assay), and probable NMDAR-IgG+ AIE in the population, as defined in Table 3. Criteria for probable NMDAR-IgG+ AIE have been published (1), showing high sensitivity and specificity in adults and children (93–95).

**Table 3:** Eligibility criteria for NMDAR-IgG+ and LGI1-IgG+ AIE.

**Diagnosis of NMDAR-IgG+ AIE**	
**6 major groups of symptoms in NMDAR-IgG+ AIE**	
Abnormal (psychiatric) behavior or cognitive dysfunction Seizures Speech dysfunction (pressured speech, verbal reduction, or mutism) Movement disorder, dyskinesias, or rigidity/abnormal postures Decreased level of consciousness Autonomic dysfunction or central hypoventilation	
**Diagnosis of definite NMDAR-IgG+ AIE**	**Diagnosis of probable NMDAR-IgG+ AIE**
Rapid onset (<3 months) of one or more of the six major groups of symptoms	Rapid onset (<3 months) of four or more of the six major groups of symptoms *or* three of the six major groups of symptoms accompanied by a systemic teratoma
Documented history or presence at screening of NMDAR-IgG antibodies in the CSF by a CBA	One of the following laboratory results: Abnormal EEG CSF with pleocytosis or oligoclonal bands
Reasonable exclusion of alternative causes^a^	Reasonable exclusion of alternative causes^a^
**Diagnosis of LGI1-IgG+ AIE**	
Documented history or presence at screening of LGI1-IgG (in serum or CSF) by a CBA	
Subacute onset (progression 4 months) of: Working memory deficits, *or* Seizures (including facio-brachial dystonic seizures), *or* Psychiatric symptoms suggesting involvement of the limbic system	
Reasonable exclusion of alternative causes^a^	

^a^Alternative causes include, but are not limited to, Bickerstaff’s brainstem encephalitis, acute disseminated encephalomyelitis, Hashimoto’s encephalopathy, primary angiitis of the CNS, Rasmussen’s encephalitis.

AIE, autoimmune encephalitis; CBA, cell-based assay; CNS, central nervous system; CSF, cerebrospinal fluid; EEG, electroencephalogram; LGI1-IgG(+), leucine-rich glioma-inactivated 1 immunoglobulin G (antibody-positive); NMDAR-IgG(+), *N*-methyl-d-aspartic acid receptor immunoglobulin G (antibody-positive).

Clinically, adolescents with AIE present similarly to adults and use near-identical treatment regimens (21, 56, 96, 97); therefore, the study aims to recruit adolescent participants in a proportion that is representative of the real-world AIE population. CIELO will investigate a potential long-term therapy for AIE in a pediatric population (Table 1) and has the potential to provide novel insight into the treatment of pediatric AIE. However, there are well-documented increased challenges in the recruitment and retention of adolescent participants to a clinical trial, such as engaging with pediatric specialists, continuity of care, and disruptions to habitual activities (98, 99). Accordingly, this study has identified and included specific sites that routinely care for children, and specific study training materials have been developed with the aim of optimizing recruitment and retention of adolescents.

#### Cohort 2: LGI1-IgG+ AIE

Eligible participants for the LGI1-IgG+ AIE cohort will be ≥18 years old with a diagnosis of LGI1-IgG+ AIE according to clinical criteria adapted from Graus et al. (1) (Table 3). Given the extremely rare occurrence of LGI1-IgG+ AIE in children (100), no adolescents will be included in this cohort.

#### Study-wide eligibility criteria

Eligible participants must meet the definition of new onset or incomplete responder AIE, as defined in Table 2. The study aims to recruit both populations in representative proportions. Other key inclusion criteria include: (1) the onset of AIE symptoms ≤9 months before randomization, to ensure that patients who have an active state of inflammatory disease are enrolled, as opposed to patients who may have temporal atrophy, encephalomalacia, and long-term sequelae as a consequence of previous AIE episodes, and (2) a stable (≥24 h) mRS score of ≥2 at study baseline (Part 1, Week 0, pre-dose assessments). Patients with mRS scores of ≥2 will be included to ensure representation from patients whose deficits, such as cognitive deficits, persist despite prior treatment and “mild” mRS scores.

After providing informed consent, participants will enter a screening period of up to 28 days. For participants otherwise unable to give consent due to the severity of their disease, informed consent may be given by a legally authorized representative (or equivalent under local law) and participant assent obtained, as per local requirements.

Exclusion criteria were selected to ensure the robustness of NMDAR-IgG+ and LGI1-IgG+ AIE diagnoses, where risk of bias related to concurrent neoplastic conditions or alternative autoantibodies or neuroimmunological conditions is eliminated. These include untreated teratoma or thymoma at study baseline, history of carcinoma or malignancy with recurrence within ≤5 years before screening, known positivity to an intracellular antigen with high cancer association, any other cell-surface neuronal antibodies other than NMDAR-IgG and LGI1-IgG in the absence of NMDAR-IgG or LGI1-IgG positivity, paraneoplastic encephalitis, history of negative anti-NMDAR antibody in CSF using a cell-based assay within 9 months of symptom onset (NMDAR-IgG+ AIE cohort), evidence of diseases that may preclude participation (e.g., central or peripheral nervous system demyelinating disease, alternative cause of associated symptoms, uncontrolled concomitant disease, and infections), and prior treatment with IL-6 inhibitor therapy, anti-CD19 antibody, complement inhibitors, neonatal Fc receptor antagonists, anti-B-lymphocyte stimulator monoclonal antibody, T-cell-depleting therapies, or investigational agents (within 24 weeks prior to screening or within five half-lives of the investigational drug, whichever is longer). To maximize recruitment, participants who fail screening may qualify for rescreening at the investigator’s discretion.

### Study treatment

All participants will have started their acute first-line therapy prior to randomization. Participants meeting the definition of incomplete responder may continue to maintain stable doses of background IST throughout the course of the study. To help reduce variability and to facilitate study interpretability, permitted background ISTs are limited to one of either mycophenolate mofetil, intravenous cyclophosphamide, or azathioprine. For incomplete responders who have previously received rituximab, a treatment course must have been initiated ≥2 months prior to screening and the last dose of rituximab must have been administered ≥4 weeks prior to randomization.

After acute first-line therapy, many experts recommend treatment with glucocorticoids, typically at a dose of >20 mg/day. Long-term glucocorticoid use for disease control (usually 3–12 months) is common in AIE, particularly LGI1-IgG+ AIE, and is associated with toxicity, adverse events (AEs), and health risks (28, 101–103). Therefore, participants who receive oral or intravenous glucocorticoids at study baseline are required to taper off using a standard taper schedule, starting 4 weeks after randomization (Week 4), unless they are in a critical-care setting. Because patients with AIE may relapse when reducing their glucocorticoid dose (28, 101), the ability to successfully taper glucocorticoids will be an important outcome of this study.

#### Study treatment: Part 1

During Part 1, participants will receive placebo or satralizumab via subcutaneous (SC) injection at Weeks 0, 2, 4, and then every 4 weeks (Q4W) until the end of Part 1. Part 1 consists of a double-blinded treatment period of 52 weeks, with primary and secondary endpoint evaluation at Week 24. The 24 weeks after initiation of satralizumab represent the period after the acute symptomatic first stage, where recovery would be expected to occur, and long-term prognosis would become evident for newly diagnosed patients. Early response to treatment is correlated with overall clinical improvement and long-term functional outcome improvements in NMDAR-IgG+ and LGI1-IgG+ AIE (46, 55). A 24-week period is expected to capture early response to treatment in both the new onset and the incomplete responder populations and determine the need for any additional lines of therapy. However, some deficits, such as cognitive impairment, may persist for more than a year (44, 104). Therefore, a 52-week double-blinded treatment period was selected to characterize the efficacy of satralizumab on longer-term outcomes of cognition, quality of life measures, and regaining of daily functioning.

A common concern reported in AIE trials is the possibility of being randomly assigned to placebo (82), particularly if participants are required to discontinue all prescribed medications. Considering that AIE can have an aggressive course, all participants are permitted to receive medications that are commonly used for the symptomatic management of AIE, as described by Abboud et al. (28). Dose decreases of symptomatic treatments will only be permitted for safety reasons during the first 24 weeks of Part 1. Additionally, participants classified as incomplete responders are permitted to continue receiving the aforementioned ISTs. Rescue therapy with commonly used medications may be administered to participants who do not improve or worsen during the course of the study, in line with current clinical management practice. Introduction of a new IST, dose increases, change of background treatment, or inability to taper off glucocorticoids due to failure to improve or worsening of disease will be treated as a rescue medication event. The increase in glucocorticoid dose or failure to taper off glucocorticoids according to the protocol-directed taper will be regarded as rescue unless prespecified criteria are met.

#### Study treatment: Part 2

After completing Part 1 of the study, participants can enter Part 2. The long-term consequences of AIE are thought to be critically influenced by treatment in the acute phase and subsequent months (21, 25, 55, 105). However, the benefit of immunomodulation after acute therapy and the optimal duration of treatment are poorly understood. Hence, Part 2 is key to the provision of longer-term data on the risks and benefits of satralizumab in AIE, as well as the generation of robust data on longer-term outcomes and treatment in AIE.

As a requirement to commit to an extension period at enrollment may negatively influence recruitment and retention of participants, an innovative approach is used for Part 2 that is designed to assist in retaining as many participants as possible in a longer-term assessment. Participants can choose a long-term treatment plan that is most suited to their individual clinical response and circumstance. Participants may choose to (1) continue on randomized treatment, (2) initiate open-label satralizumab (participants will continue to receive satralizumab SC Q4W if they were randomly assigned to satralizumab in Part 1 or receive satralizumab per the dosing regimen in Part 1 if they were randomly assigned to placebo), or (3) stop study treatment and continue with follow-up assessments.

Participants may be tapered off any remaining steroids or ISTs at the discretion of the investigator. This is allowed only after Week 12 of the extension period for participants choosing option 2 to ensure that participants treated with placebo in Part 1 have reached steady-state levels of satralizumab prior to glucocorticoid/IST taper. Satralizumab can be self-administered by participants (or their caregivers) who choose option 1 (from Week 0 of the extension period) and option 2 (from Week 24 of the extension period) on days that do not require additional assessments at the site, if deemed appropriate by the investigator. A telephone interview after each satralizumab dose will confirm treatment adherence and evaluate any changes in health status (e.g., new or worsening neurological symptoms or any possible AEs). The option to stop all study treatment is open to all participants at any time during Part 2. In addition, the option to start open-label satralizumab (option 2) is available to participants who chose to continue their original treatment plan (option 1) at any time.

Part 2 was designed to reflect the real-world choices made after receiving acute therapy for AIE. Incorporating patient preference will hopefully maximize the likelihood of patient participation in the extension period and provide insights into long-term outcomes in AIE.

#### Study assessments and endpoints

The choice of appropriate study endpoints has been a significant challenge in AIE trial development (82). For a Phase 3 study, it is important to determine clinically relevant endpoints that have clinical validity and are also sensitive enough to measure the benefit-to-risk profile of an investigational treatment. For AIE, endpoint categories include general measures of disability/functional status, cognitive assessments, and autoimmune encephalitis-focused categorical rating scales. CIELO was designed to use the mRS, a measure that covers the broad range of symptoms that are applicable in both NMDAR-IgG+ and LGI1-IgG+ AIE.

Some previous studies have used the Clinical Assessment Scale in Autoimmune Encephalitis (CASE) scores, clinical worsening (patient or clinician observations, Lawton and Brody Instrumental Activities of Daily Living Scale [IADL]), Texas Functional Living Scale (TFLS), hospitalization (for symptoms of encephalitis), or seizure count measures as the primary endpoint (Table 1) (29, 82, 85, 88, 89); however, these are associated with some limitations. Despite widespread use in AIE clinical trials, CASE has fewer data supporting its applicability for longer-term follow-up and subtler sequelae (53, 106). CASE does not incorporate categories for death, sleep dysfunction, or autonomic dysfunction (13), and there are limited data for its use in pediatric populations (107). As for seizure count, due to inadequate measures of acute seizure reporting available and the fact that not all patients experience seizures, larger sample sizes would be required to demonstrate a clinical effect when measuring clinical worsening or seizure counts as a primary endpoint.

CIELO will use the mRS as the primary outcome measure of both the NMDAR-IgG+ and LGI1-IgG+ AIE basket study arms to evaluate the benefit of satralizumab on clinical status and disability. mRS measures overall disability and has been used in AIE studies for patients with both NMDAR-IgG+ and LGI1-IgG+ subtypes with proven validity and reliability (21, 84, 87, 108, 109) (Table 1). Historically, the mRS has also been used as an outcome measure for infectious encephalitis (110), including as a primary outcome measure in herpes simplex encephalitis studies (111). Available data on the mRS in AIE have been used to help inform the CIELO study design, particularly regarding expected changes in the placebo arm.

As an endpoint that measures disability and patients’ ability to carry out activities of daily living, the mRS may help provide a more comprehensive assessment of therapeutic efficacy than seizure counts or cognitive scoring in patients with NMDAR-IgG+ or LGI1-IgG+ AIE. To enhance the reproducibility and robustness of data, a Structured Interview for the mRS (mRS-SI), adapted from Wilson and Hareendran (112), will supplement the mRS, and the mRS-SI will be tailored to evaluate specific aspects of disability and dependence associated with AIE. A selection of other clinically meaningful patient-reported outcome (PRO), performance-based outcome (PerfO), and clinician-reported outcome endpoints have also been chosen to assess the treatment benefit of satralizumab in CIELO.

#### Efficacy and safety endpoints, assessments, and analyses

The primary efficacy endpoint in CIELO is the proportion of participants with a ≥ 1-point improvement from study baseline in the mRS score and no use of rescue therapy at Week 24 (Table 4). A 1-point change in mRS score is considered to be clinically meaningful based on the severity range covered by the scale grade (113, 114) and is used in clinical practice and observational studies to demonstrate a meaningful change in activities of daily living. Because eligible participants for this study will be impaired in carrying out activities at study baseline (as measured by mRS score ≥ 2), a ≥ 1-point improvement, rather than an mRS score of 0–2, which has been defined as a good outcome in some previous studies (21, 108), will be considered to be clinically significant and meaningful (113, 114) in all participants with differing levels of symptomatology and degree of disability.

**Table 4:** Study endpoints.

Part 1 (primary treatment period)	
Primary efficacy endpoint	Proportion of participants with mRS score improvement ≥1 from study baseline and no use of rescue therapy at Week 24
Secondary efficacy endpoints^a^	Time to mRS score improvement ≥1 from study baseline without use of rescue therapy Time to rescue therapy Proportion of participants with sustained seizure cessation^b^ at Week 24, and no use of rescue therapy Change in CASE score from study baseline at Week 24 MoCA total score at Week 24 RAVLT score at Week 24 (LGI1-IgG+ AIE cohort) mRS score at Week 24 (as measured on a 7-point scale; NMDAR-IgG+ AIE cohort)
Safety endpoints	Incidence, seriousness, and severity of AEs Change from study baseline in targeted vital signs, clinical laboratory test results, ECG results, weight, height (<18 years only), and C-SSRS
Exploratory endpoints	Degree of disability, clinical severity, mood, quality of life and functional living, based on ClinRO, PerfO, and PRO scales (mRS, CASE, MoCA, RAVLT, BDI-II, EQ-5D-5L and MFIS scores) (change from study baseline and absolute scores) Additional exploratory biomarker assessments, including longitudinal assessments
Pharmacokinetics and pharmacodynamics	Serum IL-6 and soluble IL-6R Serum and/or CSF concentrations of satralizumab

^a^Not in hierarchical order; will be tailored to the individual cohort.

^b^Seizure cessation defined as cessation of seizures for 4 consecutive weeks.

AE, adverse event; AIE, autoimmune encephalitis; BDI-II, Beck Depression Inventory, second edition; CASE, Clinical Assessment Scale in Autoimmune Encephalitis; ClinRO, clinician-reported outcome; CSF, cerebrospinal fluid; C-SSRS, Columbia-Suicide Severity Rating Scale; ECG, electrocardiogram; EQ-5D-5L, EuroQol 5-Dimension 5-level; IL-6, interleukin-6; IL-6R, interleukin-6 receptor; LGI1-IgG+, leucine-rich glioma-inactivated 1 immunoglobulin G antibody-positive; MFIS, modified Fatigue Impact Scale; MoCA, Montreal Cognitive Assessment; mRS, modified Rankin Scale; NMDAR-IgG+, *N*-methyl-d-aspartic acid receptor immunoglobulin G antibody-positive; PerfO, performance-based outcome; PRO, patient-reported outcome; RAVLT, Rey Auditory Verbal Learning Test.

As AIE can have an aggressive course, rapid symptom resolution is critical in preventing potentially permanent residual long-term morbidity and mortality. The need for rescue or repeated rescue therapy, due to failure to improve or worsening of AIE-related symptoms, is indicative of unsatisfactory outcomes. Therefore, when treated with satralizumab, the lack of need for rescue therapy is a key factor suggesting its treatment benefit. Freedom from rescue therapy use, combined with improvements in disability and dependence on the mRS scale in the primary endpoint, will provide an overall clinically relevant measure of an early and durable response.

Although it was ultimately decided not to incorporate the CASE scale as a primary efficacy endpoint, it is a promising tool for evaluating the severity of AIE. The CASE score is positively associated with the mRS (115) and is more representative of the diverse symptomatology of AIE than the mRS (13). Hence, it will be assessed as a secondary efficacy endpoint (Table 4). Other secondary and exploratory efficacy endpoints will assess long-term neurological PROs and PerfOs using the Montreal Cognitive Assessment (MoCA); Rey Auditory Verbal Learning Test (RAVLT); Beck Depression Inventory, second edition (BDI-II); EuroQoL 5-Dimension 5-Level; and Modified Fatigue Impact Scale (MFIS) scores (Table 4), many of which have been used in studies to assess long-term outcomes in patients with AIE (7, 116–118). Due to the global nature of the study, PRO instruments have been translated into local languages to facilitate their use. Additionally, seizure type, frequency, duration, and severity will be captured using a patient/caregiver completed seizure diary, and electroencephalogram assessments will be conducted at select time points in Part 1 of the study. Together, these assessments will provide a global understanding of disease severity and response to treatment.

Safety endpoints on incidence, seriousness, and severity of AEs, as well as change from study baseline in targeted vital signs and laboratory tests, will be assessed (Table 4). To characterize the efficacy of satralizumab treatment on longer-term outcomes, exploratory efficacy endpoints will evaluate the durability of response to satralizumab, time course of efficacy, and time to disease improvement (Table 4).

Efficacy and safety measures will be collected throughout Part 1 and Part 2 at prespecified timepoints. Further subgroup analyses will be performed for participants with probable NMDAR-IgG+ AIE and for adolescent participants.

#### Laboratory and biomarker analyses

To explore the PK, PD, and immunogenicity of satralizumab, serum, plasma, blood, and CSF samples, as appropriate, will be taken prior to each study drug administration at prespecified time points. PK sampling will be used to analyze the impact of a range of covariates on exposure (e.g., sex, race, age, and body weight), and the relationships between exposure and PD, efficacy, immunogenicity, and safety endpoints to support the recommended dose of satralizumab in the NMDAR-IgG+/LGI1-IgG+ AIE population. PD sampling will assess target engagement in response to satralizumab. Immunogenicity sampling will assess antidrug antibodies.

Biomarker analyses will explore whether satralizumab is binding to its intended target (IL-6R) and will help to elucidate the mechanism of action of any underlying treatment effect and disease pathophysiology. These analyses may help to identify biomarkers that are predictive of a response to satralizumab, prognostic biomarkers that are associated with progression to a more severe disease state, and biomarkers associated with susceptibility to developing AEs (that could potentially lead to improved AE monitoring). Exploratory biomarker research may include analysis of NMDAR-IgG and LGI1-IgG, markers of inflammation, immune cell subset activity, CNS damage, and blood samples for flow cytometry of immune cell subsets and T-, B-, and natural killer cells. CSF sampling is included as an optional study activity at prespecified timepoints in Part 1. While lumbar puncture is routine in the diagnostic workup of AIE, research-based lumbar punctures may not be feasible for all patients and may add a potential barrier to recruitment and retention. Therefore, study lumbar punctures have been made optional.

#### Statistics

The CIELO study will compare satralizumab with placebo in approximately 152 participants with NMDAR-IgG+ or LGI1-IgG+ AIE, as defined by the study eligibility criteria. The NMDAR-IgG+ and LGI1-IgG+ AIE cohorts will be treated as separate populations and analyzed separately. Each cohort will have independent Type I error control at a two-sided 5% significance level. For adolescent participants, descriptive subgroup analyses will be performed and compared with data collected in adults to check for consistency in any observed treatment effect, and that adolescent data are comparable to the data obtained from the overall population.

Efficacy and safety analyses will be performed in the intent-to-treat and safety populations, respectively. The primary comparison of interest in each cohort is the difference between the placebo and satralizumab groups in the proportion of participants who achieve the primary endpoint, which will be tested using a Cochran–Mantel–Haenszel test by randomization stratification factors (patient population: new onset versus incomplete responders, and region: North America/Europe versus Asia versus rest of world).

Secondary efficacy endpoints will follow a cohort-specific hierarchy, allowing analyses to be tailored to the predominant clinical features and longer-term impairments in the NMDAR-IgG+ and LGI1-IgG+ AIE populations. An independent data monitoring committee will be used for data review during Part 1.

## Discussion

CIELO, a Phase 3, randomized, double-blind, basket study in patients with NMDAR-IgG+ or LGI1-IgG+ AIE, will investigate the efficacy and safety of satralizumab compared with placebo. There is a need for efficacious and well-tolerated AIE therapies that can shorten disease duration, achieve a more complete and longer-lasting response, decrease long-term impacts, and improve quality of life. However, the challenges inherent in designing and executing rare disease clinical trials have impacted the generation of prospective and robust evidence in this condition.

CIELO has been designed to reflect the unmet need of patients with AIE in a real-world clinical setting, using a primary endpoint that is a global measure of neurological function and requires a marked change in clinical status. The study design of CIELO has been informed by the challenges faced in previous and ongoing AIE studies (Table 1) (29, 82, 84–90). Specifically, CIELO will maximize (1) the identification of eligible participants, (2) the inclusion of newly treated patients (new onset) and patients treated with agents beyond first-line therapy (incomplete responder), (3) confidence with a placebo-controlled design, and (4) data generation and interpretability of study results.

CIELO will recruit participants across approximately 85 sites in 15 countries. Initiatives to increase awareness of AIE and the study are also underway, including the development of educational materials for patients and physicians, and establishing referral pathways with antibody-testing laboratories (patients who test positive for NMDAR/LGI1 autoantibodies will receive information on the CIELO study).

Because there is a clear unmet need in both newly treated (new onset) and refractory (incomplete responder) patient populations, the inclusion of both populations should help to shed further light on the utility of satralizumab, allow for a balanced representation of the real-world AIE population, and potentially make the study findings more generalizable to real-world care.

AIE can be an aggressive disease, and the possibility of being randomly assigned to placebo is a common concern reported in AIE trials (82). CIELO was designed toward increasing participant confidence because participants enrolled after receiving standard first-line and/or second-line therapy can choose to continue background ISTs and symptomatic treatments in addition to receiving rescue therapy when needed after enrollment (which reflects AIE management in real-world clinical practice). Furthermore, incorporating patient choice in Part 2 of the study will allow participants to choose a long-term treatment plan that is most suited to their needs and to maximize patient retention.

CIELO has taken the following steps to maximize data generation and interpretability of the study results: (1) the study population is restricted to NMDAR-IgG+ or LGI1-IgG+ subtypes to minimize heterogeneity; (2) the basket study design enables cohort-specific hierarchies to allow analyses to be tailored per NMDAR-IgG+ and LGI1-IgG+ subtype; (3) the mRS score, which has previously been used in AIE, as well as in infectious encephalitis studies, will be the primary endpoint; and (4) the doses of select background medications are kept stable until the primary endpoint timeline.

Although efforts have been taken to maximize the extent of investigations in the CIELO study, there are limitations inherent in the design of every clinical trial. CIELO includes a subset of participants with probable NMDAR-IgG+ AIE, for which consensus criteria (1) have shown high sensitivity and specificity in adults and children (93–95). The inclusion of this subgroup was intended to improve generalizability and practicality, particularly where CSF sampling for NMDAR-IgG may be inconsistent, delayed, or difficult to achieve. The study population with probable NMDAR-IgG+ AIE is expected to be low and sensitivity analyses will be conducted. MRI findings can be heterogenous in NMDAR-IgG+ and LGI1-IgG+ AIE (119, 120), and although exploratory neuroimaging outcomes, including lesional as well as volumetric measures, were seriously considered, research neuroimaging was ultimately not included as a study activity in the final protocol because of the lack of reliable and validated approaches for analyzing neuroimaging data in AIE. There is also emerging literature on sleep disorders in AIE (121, 122), and future trials may consider the inclusion of actigraphy or related measures as an exploratory outcome.

CIELO will investigate the efficacy and safety of IL-6 signaling inhibition with satralizumab, potentially offering a novel approach for the management of AIE and a greater understanding of the disease course. By anticipating the challenges commonly associated with AIE trials, it is hoped that this study will yield prospective, robust evidence that is currently lacking in the field. The successful implementation of this trial will contribute to the development of evidence-based medicine for AIE, ultimately benefiting both patients and their families who are affected by this condition.

## Ethics and dissemination

The study is being performed in compliance with the International Conference on Harmonization, in accordance with Good Clinical Practice guidelines, and in line with the principles of the Declaration of Helsinki and applicable local, regional, and national laws. All participants are required to provide written informed consent before any study-related procedures, including screening evaluations, are performed. Institutional review board approval will be obtained at each participating center before activating each study site.

The study was prospectively registered on ClinicalTrials.gov prior to enrolling the first participant (ClinicalTrials.gov identifier: NCT05503264). Results of this study will be reported periodically, as data become available, at international congresses where AIE is of interest to the audience and published in international peer-reviewed journals.

## Author contributions

S-TL: Writing – original draft, Writing – review & editing, Investigation, Resources, Conceptualization. HA: Writing – original draft, Writing – review & editing, Investigation, Resources, Conceptualization. SI: Writing – original draft, Writing – review & editing, Investigation, Resources, Conceptualization. HN: Writing – original draft, Writing – review & editing, Investigation, Resources, Conceptualization. AP: Writing – original draft, Writing – review & editing, Investigation, Resources, Conceptualization. SP: Writing – original draft, Writing – review & editing, Investigation, Resources, Conceptualization. EY: Writing – original draft, Writing – review & editing, Investigation, Resources, Conceptualization. JW: Writing – original draft, Writing – review & editing, Investigation, Resources, Conceptualization. SR: Writing – original draft, Writing – review & editing, Investigation, Resources, Conceptualization, Methodology, Visualization. JO: Writing – original draft, Writing – review & editing, Investigation, Resources, Conceptualization, Methodology, Visualization. JS: Writing – original draft, Writing – review & editing, Investigation, Resources, Conceptualization, Methodology, Visualization. JSL: Writing – original draft, Writing – review & editing, Investigation, Resources, Conceptualization, Methodology, Visualization. ME-K: Writing – original draft, Writing – review & editing, Investigation, Resources, Conceptualization, Methodology, Visualization. MG: Writing – original draft, Writing – review & editing, Investigation, Resources, Conceptualization, Methodology, Visualization. JG: Writing – original draft, Writing – review & editing, Investigation, Resources, Conceptualization.
